# Fibrin Clot Structure and Platelet Aggregation in Patients with Aspirin Treatment Failure

**DOI:** 10.1371/journal.pone.0071150

**Published:** 2013-08-19

**Authors:** Søs Neergaard-Petersen, Ramzi Ajjan, Anne-Mette Hvas, Katharina Hess, Sanne Bøjet Larsen, Steen Dalby Kristensen, Erik Lerkevang Grove

**Affiliations:** 1 Department of Cardiology, Aarhus University Hospital, Aarhus, Denmark; 2 Division of Cardiovascular and Diabetes Research, Leeds Institute for Genetics, Health and Therapeutics, University of Leeds, Leeds, United Kingdom; 3 Department of Clinical Biochemistry, Aarhus University Hospital, Aarhus, Denmark; 4 Department of Internal Medicine, Cardiology, University Hospital Aachen, Aachen, Germany; University of Colorado Denver, United States of America

## Abstract

**Background:**

Aspirin is a cornerstone in prevention of cardiovascular events and modulates both platelet aggregation and fibrin clot formation. Some patients experience cardiovascular events whilst on aspirin, often termed aspirin treatment failure (ATF). This study evaluated both platelet aggregation and fibrin clot structure in patients with ATF.

**Methods:**

We included 177 stable coronary artery disease patients on aspirin monotherapy. Among these, 116 (66%) had ATF defined as myocardial infarction (MI) whilst on aspirin. Platelet aggregation was assessed by Multiplate® aggregometry and VerifyNow®, whereas turbidimetric assays and scanning electron microscopy were employed to study fibrin clot characteristics.

**Results:**

Enhanced platelet aggregation was observed in patients with ATF compared with non-MI patients following stimulation with arachidonic acid 1.0 mM (median 161 (IQR 95; 222) vs. 97 (60; 1776) AU*min, p = 0.005) and collagen 1.0 µg/mL (293 (198; 427) vs. 220 (165; 370) AU*min, p = 0.03). Similarly, clot maximum absorbance, a measure of fibrin network density, was increased in patients with ATF (0.48 (0.41; 0.52) vs. 0.42 (0.38; 0.50), p = 0.02), and this was associated with thinner fibres (mean ± SD: 119.7±27.5 vs. 127.8±31.1 nm, p = 0.003) and prolonged lysis time (552 (498; 756) vs. 519 (468; 633) seconds; p = 0.02). Patients with ATF also had increased levels of C-reactive protein (CRP) (1.34 (0.48; 2.94) and 0.88 (0.32; 1.77) mg/L, p = 0.01) compared with the non-MI group. Clot maximum absorbance correlated with platelet aggregation (r = 0.31–0.35, p-values<0.001) and CRP levels (r = 0.60, p<0.001).

**Conclusions:**

Patients with aspirin treatment failure showed increased platelet aggregation and altered clot structure with impaired fibrinolysis compared with stable CAD patients without previous MI. These findings suggest that an increased risk of aspirin treatment failure may be identified by measuring both platelet function and fibrin clot structure.

## Introduction

Rupture of an atherosclerotic plaque causes platelet aggregation and activation of the coagulation cascade with formation of thrombin prompting conversion of fibrinogen to fibrin. As a result, a platelet-rich thrombus entrapped in a cross-linked fibrin network is formed, which may result in arterial occlusion and subsequent organ damage [1], [2].

Studies have shown that altered structure of fibrin clots is associated with coronary artery disease (CAD) and myocardial infarction (MI) [3]–[8]. Furthermore, a compact clot structure is associated with cardiovascular risk factors such as diabetes and smoking [9]–[11]. Thrombotic and inflammatory markers including fibrinogen and high-sensitive C-reactive protein (hs-CRP) have independently been associated with increased risk of cardiovascular events. [12], [13]. Increased plasma levels of fibrinogen directly modulate fibrin clot properties and compromise fibrinolysis, representing one mechanism explaining the association between fibrinogen levels and cardiovascular disease. In addition, markers of inflammation may affect fibrin clot structure and platelet reactivity during antiplatelet therapy [14], [15].

Aspirin inhibits platelet aggregation and reduces the risk of cardiovascular events by approximately 25% [16], yet 8–18% of patients on secondary preventive aspirin therapy experience a recurrent vascular event within 2 years of follow-up [16]. The fact that many patients experience a cardiovascular event despite aspirin treatment is often referred to as “aspirin treatment failure” (ATF) [17], [18]. Several studies have reported a considerable inter-individual variation in the platelet response to aspirin using platelet function testing, and a comprehensive meta-analysis showed that reduced antiplatelet effect of aspirin is associated with a 4-fold risk of experiencing cardiovascular events [19].

Traditionally, studies evaluating thrombosis risk have focused on either platelet aggregation or fibrin clot formation. Thus, studies investigating both platelets and protein components of coagulation in the same individuals are scarce. Clinically, these processes are highly integrated, and inhibition of both pathways is increasingly used for treatment of thrombotic cardiovascular events [20].

Studies have shown that aspirin, besides its well-known antiplatelet properties, can also directly alter clot structure and fibrinolysis [21], [22]. Therefore, we hypothesized that in a population of stable CAD patients on aspirin monotherapy, patients with ATF would have altered fibrin clot structure and/or increased platelet aggregation. The aims of the present study were to: 1) evaluate fibrin clot properties and platelet aggregation in response to aspirin in patients with and without a history of ATF, 2) investigate the association between clot structure and platelet aggregation and 3) study the association between inflammatory markers and fibrin clot properties and platelet aggregation, respectively.

## Materials and Methods

### Study population

A total of 177 patients with stable CAD were recruited from the Western Denmark Heart Registry from November 2007 to April 2008 as previously described [23], [24]. Our aim was to provide a study population of stable CAD patients with a relatively high-risk profile. Thus, the study was designed to include about 50% patients with diabetes type 2. In our study population, 116 patients (66%) had experienced a MI whilst treated with aspirin (“aspirin treatment failure”, ATF). Patients previously diagnosed with congestive heart failure (e.g. after myocardial infarction) were only included if left ventricular systolic function were in the normal range on the most recent echocardiography. A total of 727 patients were invited by letter to participate in the study. Among these, 423 did not return the envelope or declined, and a further 127 were either not eligible or were excluded according to the criteria given below. We have previously investigated the effects of diabetes on platelet function and turnover in this population [23], [24]. In brief, patients were eligible for inclusion if they were 18 years or older and had significant CAD verified by coronary angiography showing at least 50% luminal narrowing in one or more coronary arteries, previous percutaneous coronary intervention or coronary artery bypass grafting. Patients with previous MI had suffered one or more MI verified by chest pain, electrocardiographic changes and/or elevated plasma troponin T together with raised plasma creatine kinase-MB. Exclusion criteria were a) platelet count <120×10^9^/L or b) treatment with warfarin or any drug known to affect platelet function other than aspirin (e.g. clopidogrel, NSAIDs, ticlopidin, dipyridamol) or c) ischaemic vascular events, percutaneous coronary intervention or coronary artery bypass grafting within the previous 12 months.

All participants gave written informed consent and the study was conducted in agreement with the Helsinki-II-declaration and approved by The Central Denmark Region Committees on Health Research (M-20070180).

### Compliance

All patients were taking 75 mg plain aspirin tablets daily. To optimize compliance and uniform pharmacokinetics, each patient received a pill-dispensing box with seven tablets of 75 mg non-enteric coated aspirin (Hjerdyl®, Sandoz, Denmark). All patients ingested an aspirin one hour before blood sampling. Compliance was optimized by face-to-face interviews and pill-counting, and was confirmed by measurements of serum thromboxane B_2_ (TXB_2_), which is regarded as the most specific test to evaluate the biochemical effect of aspirin on platelets [17], [25].

### Blood sampling

Blood samples were obtained at least 12 months after the acute event in order to ensure that patients were off clopidogrel therapy. Blood samples were obtained from the antecubital vein with patients in supine position after 30 minutes of rest using vacuum tubes, a large bore needle (19 G), and a minimum of stasis. Citrated plasma was centrifuged (25 min, 3300 *g*) within 30 minutes of collection, immediately frozen and stored in aliquots at −80°C. For the assessment of platelet aggregation, blood samples rested for at least 30 minutes at room temperature but no longer than 2 hours before analysis.

### Fibrin clot structure and lysis

Fibrin clot structure was evaluated by in-house turbidimetric assays in citrated plasma in duplicate as previously described [6]. The following parameters were recorded: a) Clot maximum absorbance (arbitrary units (au)) evaluating fibrin fiber thickness and network density; b) Lysis time (seconds) corresponding to the time from maximum clot formation to 50% lysis, an indicator of fibrinolytic potential; c) Lysis area, which reflects the balance between clot formation and lysis. Intra-assay and inter-assay coefficients of variation for all values were below 10%.

### Scanning Electron Microscopy

Clots were prepared in duplicates from pooled plasma from 20 randomly chosen patients with previous MI and 20 age- and sex-matched patients without previous MI. Clots were prepared and processed by stepwise dehydration as previously described [26]. All clots were prepared in duplicates and viewed and photographed at ×5,000, ×10,000 and ×30,000 magnifications using a field-emission scanning electron microscope (Quanta™ 200F FEG ESEM, FEI Company, Eindhoven, Netherlands) in 4 different areas of each clot. Fiber diameters of all clots were measured with image analysis software package ImageJ 1.45 (National Institutes of Health, USA). Fiber diameter (n = 30) from 8 separate areas of each clot was measured (total 240 fibers/fibrin clot), and comparisons were made between the different clots.

### Fibrinogen

Fibrinogen was quantified by the functional Clauss method using KC 10™ coagulometer (Henrich Amelung GmbH, Lemgo, Germany) [27].

### Platelet aggregation tests

Platelet aggregation was evaluated by 1) multiple electrode aggregometry (MEA) using Multiplate® aggregometer (Dynabyte, Münich, Germany) with arachidonic acid (AA) 1.0 mM, collagen 1.0 µg/mL and adenosine diphosphate (ADP) 10 µM as agonists and by 2) the VerifyNow® Aspirin assay (Accumetrics, CA, USA) as previously described [24]. Platelet aggregation was expressed as area under the curve (AUC, Aggregation Units×minutes (AU*min)) using Multiplate® and as Aspirin Reaction Units (ARU) using VerifyNow®.

### Platelet activation and serum thromboxane B_2_

Platelet activation was evaluated by analysis of soluble P-selectin concentrations determined by an ELISA according to manufacturer's instructions (R&D systems, MN, USA).

Serum TXB_2_ concentrations were measured according to Patrono et al. with the modification that serum was collected from whole blood by centrifugation (2600 *g* for 10 minutes) after 1 hour of clotting, and an ELISA was used (Cayman Chemical, MI, USA) [28].

### Inflammatory markers

Plasma levels of hs-CRP and complement C3 were analysed by in-house ELISAs using antibodies from DAKO (Cambridge, UK) as previously described [29]. Intra-assay and inter-assay coefficients of variation were below 5% for both assays. Leucocytes, haemoglobin and platelet count were determined by an XE-2100 hematology analyzer (Sysmex, Kobe, Japan).

### Statistical analyses

Continuous data are presented as mean and standard deviation (SD) if data were normally distributed, and as median and interquartile range (IQR) if not. For normally distributed data, a two-sided t-test was used to test the difference between two unpaired groups, whereas the Mann-Whitney test was used for data that were not normally distributed. Differences in proportions between two or more groups were evaluated using the Chi-square test or Fisher's exact test, as appropriate. Multiple linear regression analysis was used to adjust for relevant variables when comparing groups. When assessing clot structure we adjusted for age, due to the age difference between groups, fibrinogen and treatment with ACE inhibitors, diuretics and insulin. When assessing platelet activity, adjustments were also made for platelet count, since it affects measurements of platelet function [30]. Correlations between parameters were tested using Spearman's correlation. Logistic regression was employed to examine clot structure and platelet aggregation as determinants of previous MI while on aspirin, and the logistic model included age, sex, AA-induced aggregation and clot lysis area. A two-sided p-value <0.05 was considered statistically significant. Statistical analyses were performed using STATA® version 11 (StataCorp LP, TX, USA) and graphs prepared using GraphPad Prism® version 5.0 (GraphPad Software, CA, USA). Sample size was based on power calculations from previous studies published by our group [23], [24]. As previously described, the level of significance was chosen to be 5% (2-alfa) and the power to be 90% (1-beta). This required a minimum of 170 patients in total.

## Results

Clinical characteristics of the study population are shown in Table 1. Patients with previous MI whilst on aspirin were three years older than patients without previous MI, and they more frequently had a history of percutaneous coronary intervention and treatment with ACE inhibitors and insulin.

**Table 1:** Baseline characteristics of the study population (n = 177).

	CAD without MI	CAD with MI (ATF patients)	*p*-value
*Variables*	*n* = 61	*n* = 116	
Age, years	64±8	67±8	0.02
Female, n (%)	14 (23)	17 (15)	0.17
BMI, kg/m2	28±4	28±4	0.80
Systolic Blood pressure, mmHg	147±20	143±23	0.25
Diastolic Blood pressure, mmHg	85±12	85±12	0.92
Current smokers, n (%)	12 (18)	29 (25)	0.43
B-Haemoglobin, mmol/L	8.7±0.8	8.8±0.8	0.30
B-Platelet count, 10∧9/L	223 (191; 259)	234 (194; 267)	0.41
P-Creatinine, µmol/L	79 (70; 91)	81 (73; 94)	0.52
Estimated GFR, mL/min	81±23	79±21	0.61
B-Haemoglobin A1c, %	6.3 (5.7; 6.9)	6.2 (5.8; 7.2)	0.64
P-total cholesterol, mmol/L§	4.0 (3.5–4.5)	4.2 (3.6–4.9)	0.06
P-LDL cholesterol, mmol/L§	1.9 (1.7–2.2)	2.2 (1.8–2.8)	0.01
P-HDL cholesterol, mmol/L§	1.2 (1.0–1.5)	1.2 (1.0–1.4)	0.80
P-Triglycerides, mmol/L§	1.4 (1.0–1.8)	1.4 (1.0–2.1)	0.82

*Morbidity*	n (%)	n (%)	
Myocardial infarction	0 (0)	116 (100)	<0.001
By-pass surgery	14 (23)	27 (23)	0.96
Percutaneous coronary intervention	51 (84)	112 (96)	0.002
Atrial fibrillation	0 (0)	0 (0)	1.00
Coronary vessel disease (1/2/3)	31/19/10	49/31/35	0.141
Peripheral artery disease	0 (0)	3 (2.6)	0.552
Stroke	5 (8)	12 (11)	0.62
Type 2 diabetes mellitus	30 (49)	29 (47)	0.82

*Medication*			
Statins	56 (92)	99 (87)	0.32
Beta-blockers	42 (69)	91 (79)	0.13
ACE inhibitors	18 (30)	57 (50)	0.01
ARB inhibitors	9 (15)	17 (15)	0.99
Calcium antagonists	16 (26)	27 (23)	0.69
Diuretics	12 (20)	39 (34)	0.047
Proton pump inhibitors	6 (10)	11 (10)	0.95
Insulin	6 (10)	27 (23)	0.03
Any oral antidiabetic drug*	26 (43)	42 (36)	0.40
Metformin	12 (20)	21 (18)	0.80

Data expressed as mean ± standard deviation or n (%). ATF: aspirin treatment failure; GFR: Glomerular filtration rate; ACE: angiotensin-converting enzyme; ARB: angiotensin receptor blocker; B- and P- denotes whether measurements were performed in blood or plasma.

§ All blood samples were obtained on the same day, except for lipid levels, which were obtained for 114 patients from a database (measurements performed during or a maximum of 12 months prior to the study).

* Including metformin.

### Compliance

All patients returned empty pill boxes and claimed to be fully compliant with aspirin monotherapy in face-to-face interviews. In addition, compliance was confirmed by serum TXB_2_ levels below 7.2 ng/mL in all patients, which is well below 30 ng/mL that corresponds to a more than 95% inhibition of platelet cyclooxygenase-1 activity [31]. Serum TXB_2_ did not differ between non-MI patients and patients with previous MI neither before nor after adjustment for age, fibrinogen, platelet count, ACE inhibitors, diuretics, and insulin (p = 0.19 and p = 0.20, respectively).

### Patients with aspirin treatment failure (ATF) versus patients with no history of MI

**Figure 1:**
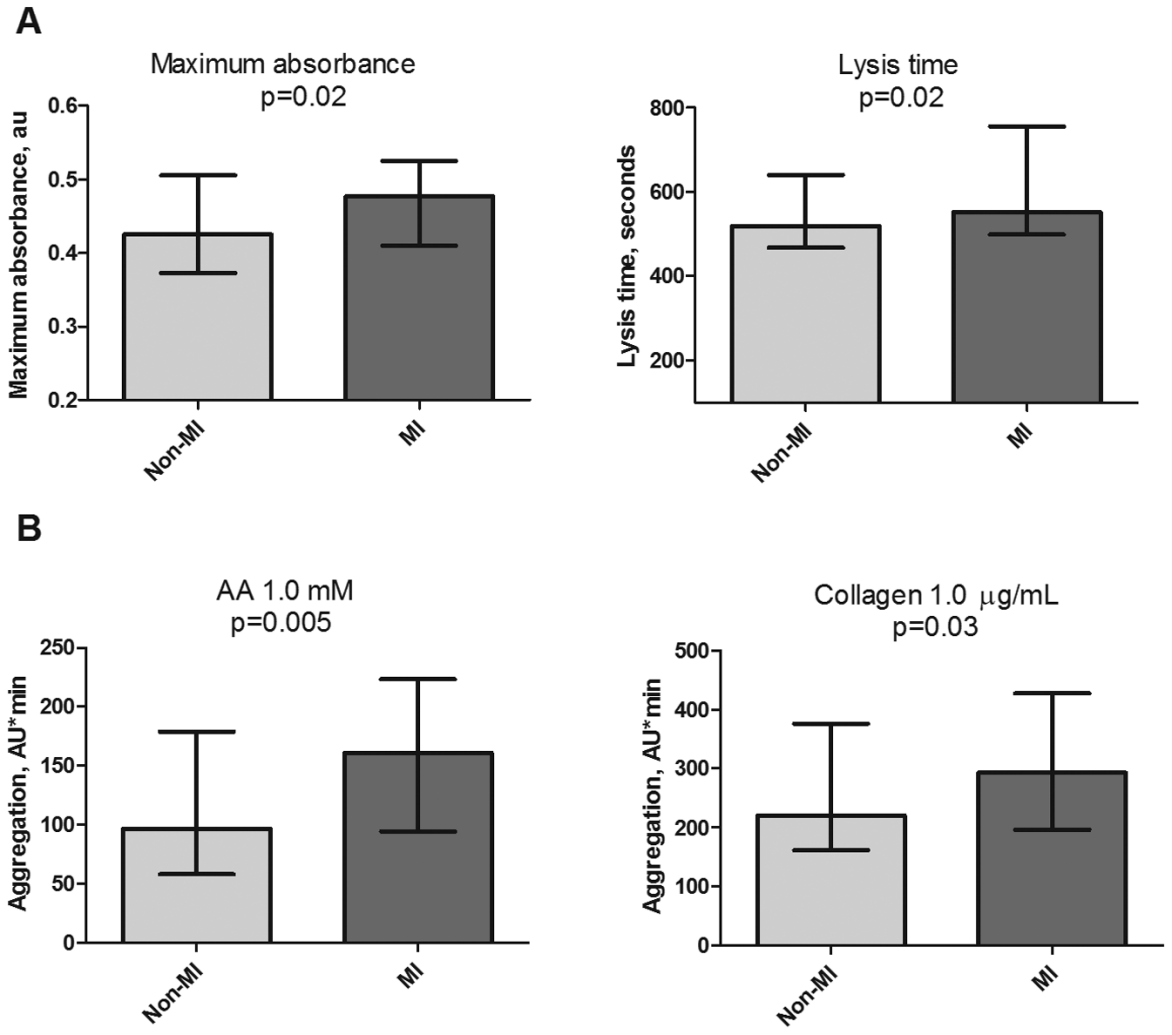
Fibrin clot structure and platelet aggregation. CAD patients without previous MI compared with CAD patients with previous MI on aspirin (ATF) assesed by turbidimetric clot assays (n = 172) and Multiplate® aggregometry (n = 174), respectively. Results are presented as median±IQR.

As shown in Figure 1A, clot maximum absorbance was increased in patients with ATF compared to patients without a history of MI (median 0.48 (IQR 0.41; 0.52) vs. 0.42 (IQR 0.38; 0.50) au, respectively; p = 0.02), confirming the presence of denser clots in patients with previous MI. Lysis time showed a similar pattern (median 552 (498; 756) vs. 519 (468; 633) sec; p = 0.02), as did lysis area (median 349 (244; 485) vs. 254 (196; 379) au, p = 0.003). Fibrinogen levels did not differ between patients with or without previous MI (median 3.8 (2.5; 4.8) and 3.5 (2.7; 5) mg/mL, p = 0.89). These findings remained unchanged after adjustment for age, fibrinogen levels, ACE inhibitors, diuretics.

**Figure 2:**
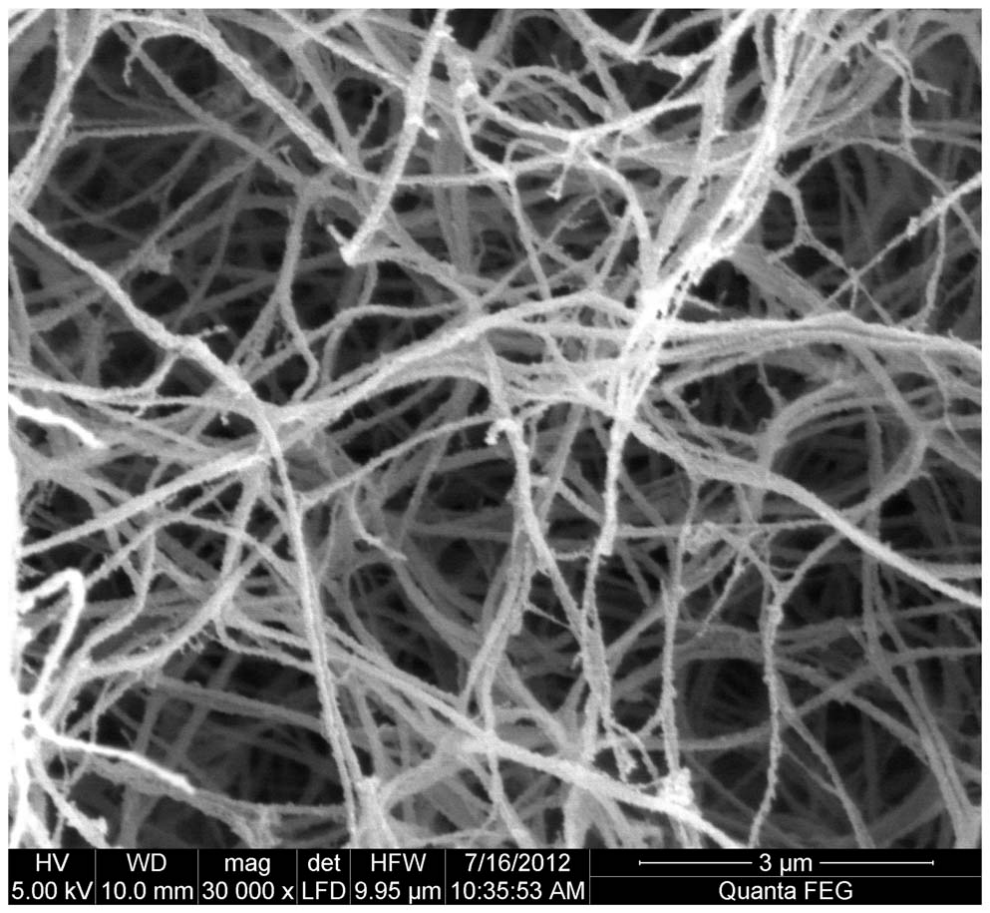
Scanning electron microscopy. Fibrin clots from age- and sex-matched patients without previous myocardial infarction using pooled plasma (n = 20 in each group). Clots shown at ×30,000 magnification.

**Figure 3:**
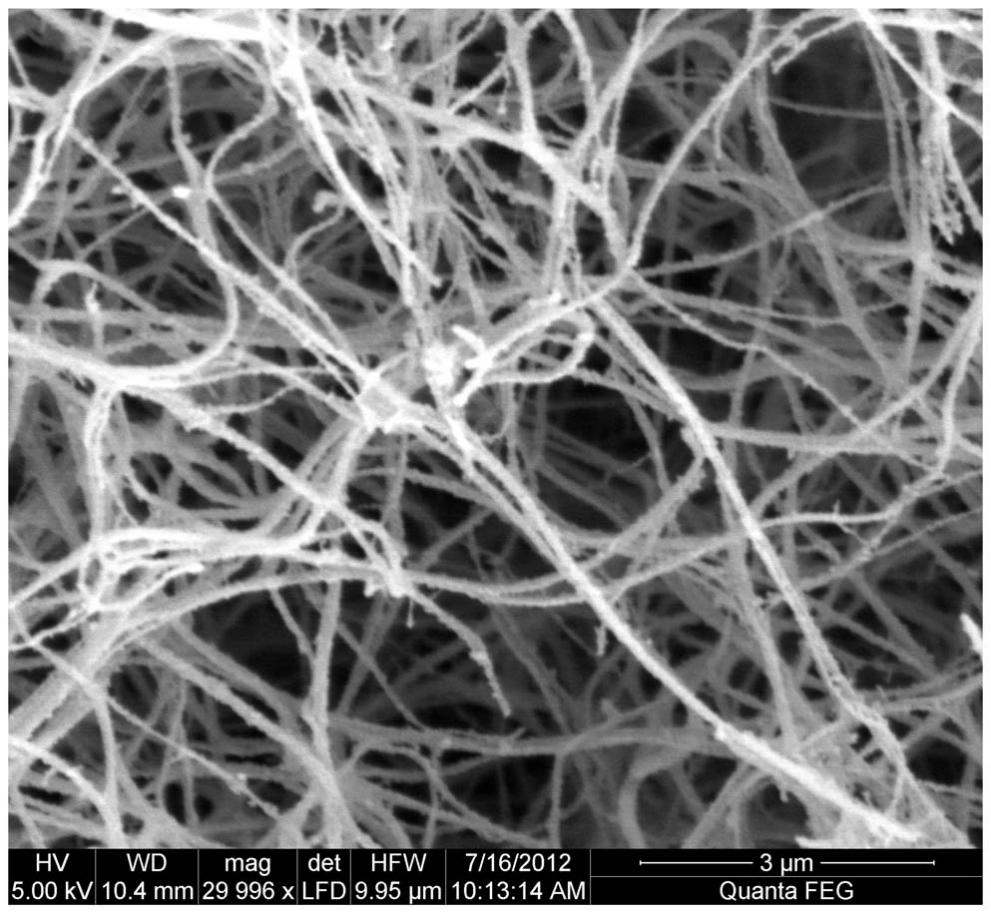
Scanning electron microscopy. Fibrin clots from age- and sex-matched patients with previous myocardial infarction on aspirin (aspirin treatment failure) using pooled plasma (n = 20 in each group). Clots shown at ×30,000 magnification.

Scanning electron microscopy (Figures 2 and 3) was consistent with more compact clots and thinner fibers in individuals with previous MI compared to those without (mean ± SD: 119.7±27.5 vs. 127.8±31.1 nm), respectively, p = 0.003).

In this population of stable CAD patients on aspirin monotherapy, patients with ATF had significantly enhanced AA- and collagen-induced platelet aggregation using MEA compared with patients without a history of MI (AA 1.0 mM median 161 (95; 222) vs. 97 (60; 1776) AU*min, p = 0.005 and collagen 1.0 µg/mL median 293 (198; 427) vs. 220 (165; 370) AU*min, p = 0.03), as shown in Figure 1B. No difference was found when ADP was used as an agonist (mean ± SD: 523±159 vs. 477±158 AU*min, p = 0.07) or with the VerifyNow® Aspirin assay (median 436 (423; 458) vs. 433 (419; 463) ARU, p = 0.70). After adjustment for age, fibrinogen, platelet count, ACE inhibitors, diuretics, and insulin, differences remained significant for AA- (p = 0.01) and collagen-induced aggregation (p = 0.02), and did not change the results for ADP-induced aggregation (p = 0.34) or VerifyNow® (p = 0.27). There was no significant difference in platelet activation evaluated by soluble P-selectin between the two groups (mean ± SD: 71±28 and 72±27 ng/mL, p = 0.88).

### Association between fibrin clot structure and platelet aggregation

As shown in Table 2, significant correlations were observed between whole blood platelet aggregation measured by MEA and both fibrinogen levels and parameters of fibrin clot structure including maximum absorbance and lysis area but not clot lysis time. Correlations between clot structure and platelet aggregation were also performed with patients divided into those with and without previous MI. This did not change the associations observed (data not shown).

**Table 2:** Correlations between fibrin clot structure parameters and platelet aggregation in patients with coronary artery disease.

	Multiple electrode aggregometry (Multiplate®)						VerifyNow®	
	AA 1.0 mM		Collagen 1.0 µg/mL		ADP 10 µM		AA	
	*r*	*p*-value	*r*	*p*-value	*r*	*p*-value	*r*	*p*-value
Fibrinogen	0.31	<0.0001	0.30	0.0001	0.24	0.001	0.13	0.08
Maximum absorbance	0.35	<0.0001	0.31	0.0001	0.33	<0.0001	0.11	0.17
Lysis time	0.13	0.10	0.05	0.55	0.11	0.17	−0.03	0.73
Lysis AUC	0.29	0.0001	0.19	0.01	0.31	<0.0001	0.04	0.56

n = 169. All data are presented with Spearmans r and p-value. AA, Arachidonic acid; ADP, Adenosine diphosphate; Lysis AUC, Lysis area under the curve.

Employing a logistic regression model including adjustment for age and sex, we examined whether clot structure and/or platelet aggregation were independent determinants of previous MI whilst on aspirin (ATF). Clot structure evaluated by lysis area (p = 0.03) and AA-induced platelet aggregation (p = 0.02) were both independent determinants of previous MI on aspirin.

### Markers of inflammation

Patients with ATF had higher leucocyte counts compared with patients without previous MI (mean ± SD: 7.6±1.9 vs. 6.8±1.7 10^9^/L, p = 0.01) as well as higher hs-CRP levels (median 1.34 (0.48; 2.94) and 0.88 (0.32; 1.77) mg/L, p = 0.01), whereas the difference in C3 levels did not reach statistical difference (mean ± SD: 0.82±0.15 vs. 0.87±0.17 mg/mL, p = 0.09).

Leukocyte count, hs-CRP and C3 showed a significant positive correlation with clot maximum absorbance (*r* = 0.30, *r* = 0.60 and *r* = 0.33, respectively, all *p*-values<0.001) and lysis area (*r* = 0.19, *r* = 0.36 and *r* = 0.20, respectively, all *p*-values<0.02), but not with lysis time (*r* = −0.03, *r* = 0.09 and *r* = 0.07, respectively, all *p*-values>0.25). A significant positive correlation was also observed for the three inflammatory markers with AA-induced aggregation (*r* = 0.45, *r* = 0.19 and *r* = 0.22, respectively, all *p*-values<0.05) and with 1.0 µg/mL collagen (*r* = 0.27, *r* = 0.17 and *r* = 0.16, respectively, all *p*-values<0.05).

## Discussion

Aspirin monotherapy is a cornerstone in prevention of cardiovascular events in patients with CAD. However, some patients experience a cardiovascular event despite aspirin treatment, often termed “aspirin treatment failure”. To our knowledge, this is the first study to evaluate the effect of aspirin on both functional fibrin clot structure properties and platelet aggregation in patients with ATF. A number of observations emerge from this study: 1) Patients with ATF were characterized by both altered clot structure with impaired fibrinolysis as well as increased AA- and collagen-induced platelet aggregation 2) Fibrin clot structure parameters and platelet aggregation showed correlation. Although statistically significant, the correlations were relatively weak. 3) Inflammatory markers were increased in patients with ATF and correlated with clot structure and platelet aggregation, suggesting an association between inflammatory and thrombotic pathways in this population.

Compliance has been implicated as an important cause of compromised antiplatelet response to aspirin [18]. All patients included in our study had serum TXB_2_ levels below 7.2 ng/mL, thus ensuring that 75 mg/day of aspirin was adhered to and ruling out compliance issues.

Inhibition of cycloxygenase-1 and, hence, thromboxane production is regarded as the primary antiplatelet mode of action for aspirin [17]. However, aspirin may affect other thrombotic pathways, which are not widely acknowledged with both *in vitro* and *in vivo* studies showing that aspirin can directly alter clot structure and enhance fibrinolysis [21], [22]. A number of fibrin clot structure measures were altered in patients with a history of MI despite aspirin treatment, including increased fibrin clot maximum absorbance and prolonged lysis time, consequently leading to larger lysis area, consistent with the formation of prothrombotic clots. These compact clots were associated with thinner fibers obtained by scanning electron microscopy and may explain, at least in part, the prolonged lysis time [32]. Although previous work has shown that patients with CAD and previous MI have a thrombotic clot profile [4], [5], this is the first study to report altered clot structure in patients with ATF. Given similar fibrinogen levels, the observed changes in clot structure parameters may be related to qualitative changes in fibrinogen or quantitative/qualitative changes in other plasma factors [12].

In addition to an altered clot structure, patients with ATF also had increased AA- and collagen-induced platelet aggregation despite aspirin treatment. This is consistent with our recent study demonstrating increased AA-induced aggregation in patients with previous MI [33]. The current work further emphasizes that individuals sustaining an MI whilst on aspirin therapy display a prothrombotic clot profile, although these findings were not confirmed using the VerifyNow® Aspirin assay. This lack of consistency may be explained by different test principles in the employed platelet tests [25], and by the fact that AA-induced platelet aggregation evaluated by MEA is more sensitive to aspirin treatment than VerifyNow® [34]. Platelet aggregation is affected by platelet count [30], but all differences between patients with and without previous MI remained after adjusting for platelet count, age, and fibrinogen. In this population of stable CAD patients on aspirin monotherapy, the increased AA- as well as collagen-induced aggregation reported in patients with previous MI suggests reduced inhibition of cyclooxygenase-1 by aspirin in patients with ATF. However, serum TXB_2_ levels were not elevated in these patients. This suggests the presence of non-COX-1 dependent mechanisms for ATF in these individuals, which remains an area for future research.

The causes of ATF may be multifactorial. A possible cause could be increased cardiovascular risk factors. In the present study, the frequency of diabetes and levels of HbA1c were similar in the two groups, but a higher percentage of patients with previous MI on aspirin were treated with insulin. This may be due to longer duration of type 2 diabetes, which is associated with higher risk of cardiovascular events [35]. In addition, patients with ATF had increased levels of leukocyte counts and hs-CRP compared with non-MI patients. In particular, increased levels of CRP have been independently associated with coronary events [13]. Inflammatory markers including leukocyte counts, hs-CRP and C3 all correlated significantly with clot maximum absorbance and AA- and collagen-induced platelet aggregation. One may hypothesize that an inflammatory plasma milieu affects fibrin clot structure and platelet aggregation. In summary, accumulating cardiovascular risk factors with insulin-treated type 2 diabetes, increased low-grade inflammation together with altered clot structure and platelet aggregation, may partly explain ATF in these patients. Alternatively, impaired acetylation of non-platelet protein, in particular fibrinogen, may explain impaired fibrin clot lysis in individuals with ATF, but this remains an area for future research.

An increased residual platelet aggregation has been associated with a 4-fold increased risk of cardiovascular events [19], and in addition, an altered clot structure characterized by impaired fibrinolysis has been associated with increased cardiovascular risk [3], [4], [6], [8] and increased cardiovascular mortality [7]. In agreement with these findings, we found that both clot structure and AA-induced platelet aggregation were independent determinants of previous MI on aspirin. As opposed to a previous study [36], we report a significant correlation between functional fibrin clot structure properties and whole blood platelet aggregation. This may indicate that denser fibrin clots being resistant to fibrinolysis are associated with increased platelet aggregation, creating a thrombotic environment. In support of this hypothesis, a denser fibrin clot structure with attenuated fibrinolysis has been shown to be influenced by platelets, including negatively charged polyphosphate as well as platelet factor-4 secreted by activated platelets [37], [38]. Taken together, our findings suggest that measurements of both fibrin clot parameters and platelet reactivity may be relevant in future studies evaluating the risk of thrombosis in aspirin-treated high-risk patients.

### Study limitations

Our data have potential clinical implications, but there are limitations to the study that should be acknowledged. Firstly, our study groups were not matched for age, but the age difference was small and the overall conclusions about clot structure and platelet aggregation remained unchanged in adjusted analyses. Secondly, no baseline measurements of clot structure and platelet function off aspirin were performed since discontinuation of aspirin was considered unethical in these high-risk patients. Thirdly, when interpreting our results it should be kept in mind, that although statistically significant, many of the correlations observed were relatively weak. Fourthly, the nature of the study did not allow the demonstration of any causality between altered clot structure/platelet aggregation during aspirin treatment and future cardiovascular events. However, our work forms the basis for future prospective studies to determine whether the observed differences in clot structure and platelet aggregation are clinically relevant.

## Conclusions

In a population of stable CAD patients on aspirin monotherapy we demonstrate both increased platelet aggregation and altered clot structure with impaired fibrinolysis in patients with previous MI whilst on aspirin treatment. Fibrin clot properties and platelet aggregation correlated, although relatively weakly, and were associated with markers of inflammation. Overall, these findings may partly explain clinical treatment failure to aspirin.
